# Subtle Crucial X-Ray Findings in Pediatric Foreign Body Aspiration

**DOI:** 10.7759/cureus.14898

**Published:** 2021-05-08

**Authors:** Akinori Sekioka, Masashi Koyama, Koji Fukumoto, Akiyoshi Nomura, Naoto Urushihara

**Affiliations:** 1 Pediatric Surgery, Shizuoka Children's Hospital, Shizuoka, JPN; 2 Radiology, Shizuoka Children's Hospital, Shizuoka, JPN; 3 Pediatric Surgery, Shizuoka Children’s Hospital, Shizuoka, JPN

**Keywords:** children, foreign body, lobar bronchus, radiolucent, lobar emphysema

## Abstract

Foreign body aspiration (FBA), with potentially life-threatening outcomes, is not unusual in the pediatric population. We report two cases of lobar bronchial radiolucent foreign bodies. Chest X-ray (CXR) showed a slight but significant finding of lobar emphysema without a significant mediastinal shift. This is possibly a key to suspecting foreign bodies. In the clinical field, a stepwise approach to detecting foreign bodies is commonly performed, from less invasive options such as CXR to computed tomography (CT). In this context, clinicians should scrupulously check CXRs when pediatric patients complain of respiratory symptoms, especially with potential FBA history.

## Introduction

Foreign body aspiration (FBA) is a relatively common but life-threatening accident in the pediatric population [1,2]. If the detection of FBA is delayed, surgical intervention may be required or, in the worst-case scenario, deadly conditions may ensue [3,4]. A definitive diagnosis of FBA is often challenging because a history of aspiration, physical examination, and radiographical investigations are frequently less evident [3,5,6].

 A chest X-ray (CXR) is a basic test for investigating FBA. Although its efficacy is still controversial, it is thought to play an important role as an initial part of the stepwise approach to detecting a foreign body [5,7-10].

This report describes two cases of FBA with unspecified symptoms and unwitnessed aspiration. CXR findings provided initial clues to the diagnosis and were vital in further evaluation and management.

## Case presentation

Case 1

**Figure 1:**
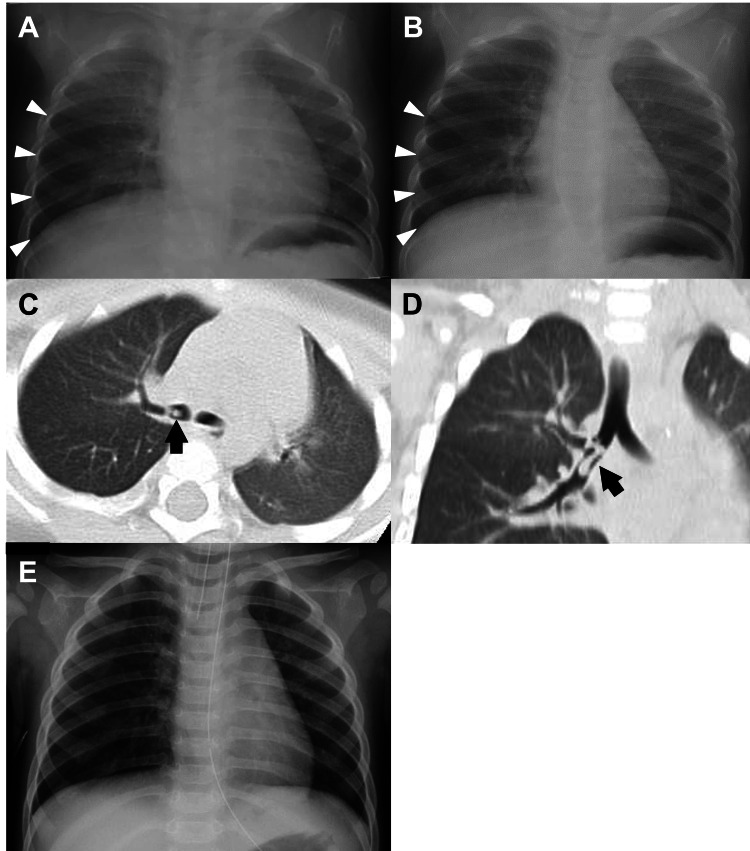
CXR showing slight emphysema of the right lower lung. (A) Expiratory phase. (B) Inspiratory phase. (C) Axial CT showing a plastic foreign body at the right inferior lobar bronchus (black arrow). (D) Coronal CT showing the seven-shaped foreign body (black arrow). (E) CXR, after removing the foreign body, in which partial emphysema disappeared. CXR, chest X-ray; CT, computed tomography

An 11-month-old girl was suspected of ingesting a part of a plastic toy. Because of parental concerns, she was taken to a private clinic the next morning with a mild wheeze. Although her general condition was good and percutaneous oxygen saturation was 98% (on room air), she was referred to a secondary referral hospital for further evaluation. A CXR showed trivial but concerning right lower lobar emphysema (Figures 1A, 1B). A subsequent chest computed tomography (CT) revealed a foreign body in the right lower lobar bronchus (Figures 1C, 1D). Based on these results, she was transferred to our tertiary hospital for foreign body removal. Flexible bronchoscopy was emergently performed under general anesthesia. Open surgery was avoided by use of alligator forceps to remove the plastic foreign body. On postoperative day (POD) 1, her respiratory status and CXR were normal (Figure 1E) and she was discharged from the hospital.

Case 2

**Figure 2:**
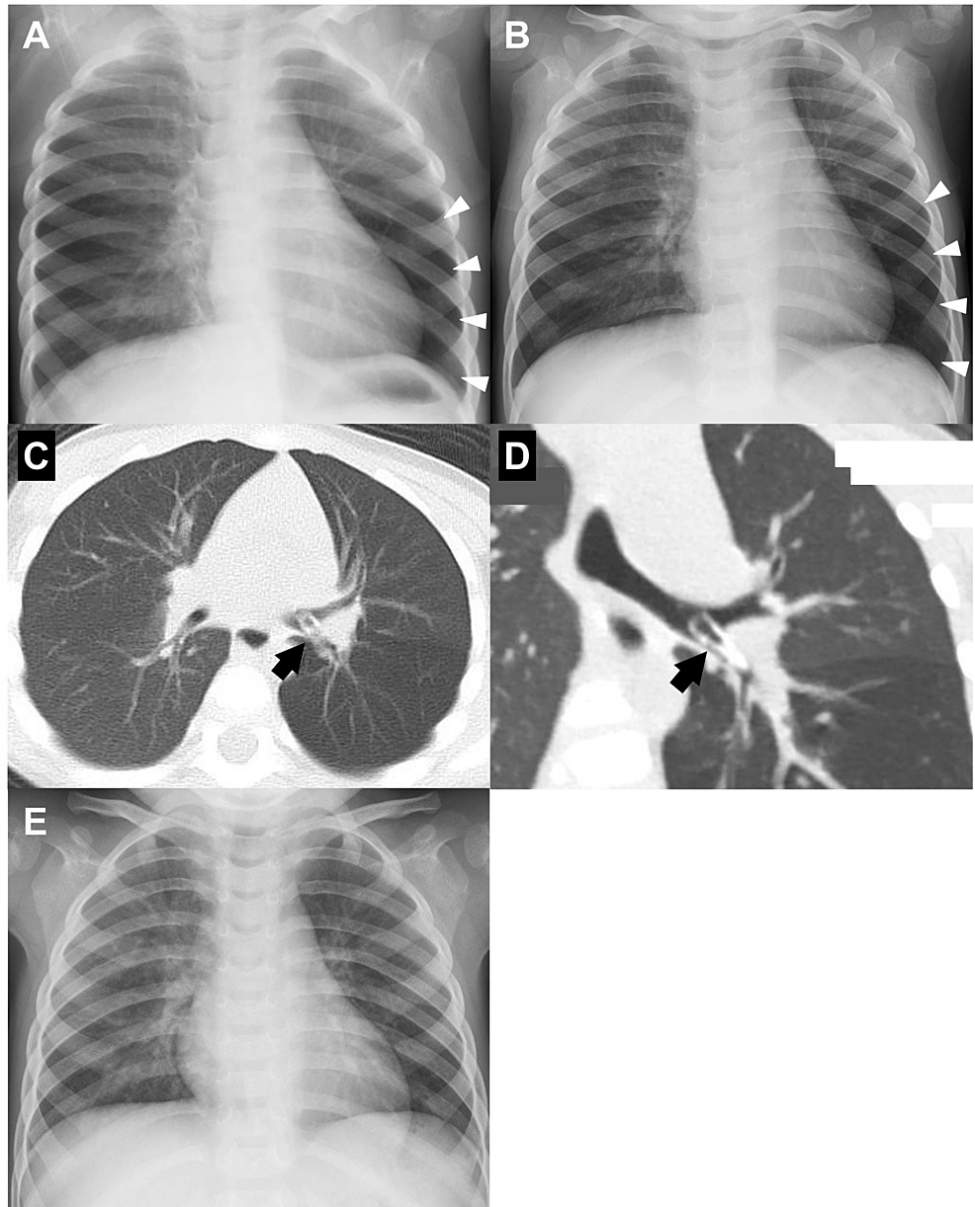
CXR showing slight emphysema of the left lower lung. (A) CXR on the first day of visiting the hospital. (B) CXR four days later. (C) Axial CT showing a plastic foreign body at the left inferior lobar bronchus (black arrow). (D) Coronal CT showing the cranial foreign body (black arrow). (E) CXR after removing the foreign body, in which partial emphysema disappeared. CXR, chest X-ray; CT, computed tomography

A 23-month-old boy presented with a slight cough and runny nose. He was taken to a secondary referral hospital and diagnosed with a viral airway infection. Oral dexamethasone, L-carbocysteine, and ambroxol were prescribed for two days. His symptoms improved, but CXR showed slight segmental emphysema in the left lower lobe. This finding led to a series of observations (Figures 2A, 2B). Further history evaluation revealed that he accidentally inserted small plastic parts of a pen into his nostrils three months prior to presentation. Subsequent CT revealed the foreign body in the left lower lobar bronchus (Figures 2C, 2D). He was then transferred to our hospital and underwent emergent flexible bronchoscopy under general anesthesia. The foreign body was partially covered with granuloma; however, it was successfully removed via alligator forceps. The postoperative course was uneventful, and CXR on POD 1 was normal (Figure 2E). He was discharged on POD 4.

## Discussion

FBA is problematic in the pediatric population, especially in younger age groups with narrow airways. Making the diagnosis is usually the main challenge, as the symptoms vary and are unspecific. Also, the act is often unwitnessed [3,5,6]. Additionally, the majority of foreign bodies are radiolucent, making the diagnosis even more difficult [8].

In the diagnostic evaluation of FBA, stepwise methods are recommended in combination with taking a careful history, physical examination, and radiological investigations [10]. Among radiological imaging modalities, CT has a high sensitivity and specificity to detect most radiolucent foreign bodies [11-13]. In contrast, some reports prioritize direct bronchoscopy over radiological detections, as CT has a limited level of detection [9,14]. Since both of these investigations are invasive, candidates should be carefully selected.

CXR is a traditional non-invasive imaging modality that is widely available and affordable. Although the significance of CXR is still controversial in the diagnostic evaluation of FBA, it can be an important predictor [4,5,8,9,11,12,15-17]. In the present cases, history and slight symptoms were unspecific clues of FBA. However, CXR showed partial emphysema without a significant mediastinal shift in the unilateral lung. This image led to further CT evaluation, which confirmed the presence of a lobar bronchial foreign body. In the initial evaluation, CXR played a significant role in predicting FBA in children.

In the face of unclear evidence of FBA, clinicians should place careful attention on CXR findings, especially unilateral partial emphysema, as shown in these cases. If possible, CXR should be taken in both inspiration and expiration in order to provide more significant information [7]. However, in younger children or infants, the timing of CXR is quite challenging because of a lack of following order. Therefore, it would be beneficial to continue follow-up of those patients, checking the symptoms and CXR repeatedly. Furthermore, recent reports showed the new tool to evaluate radiodensity on CXR, which has been expected to be useful in the diagnosis of FBA [18,19].

To the best of our knowledge, this is the first case report to demonstrate typical images of partial emphysema without significant mediastinal shift caused by FBA, which would be helpful to clinicians.

## Conclusions

We reported two cases of unilateral lobar emphysema on CXR, which is key to confirming FBA. Although CT or bronchoscopy would have been decisive in detecting FBA, performing and carefully interpreting a CXR is still important for recognizing the presence of a foreign body.
